# Collecting evidence of validity for an assessment tool for Norwegian medical students’ non-technical skills (NorMS-NTS): usability and reliability when used by novice raters

**DOI:** 10.1186/s12909-023-04837-6

**Published:** 2023-11-15

**Authors:** Katrine Prydz, Peter Dieckmann, Hans Fagertun, David Musson, Torben Wisborg

**Affiliations:** 1 Interprofessional Rural Research Team, Faculty of Health Sciences, Department of Clinical Medicine, University of Tromsø – the Arctic University of Norway, Hammerfest, Norway; 2 Hammerfest Hospital, Finnmark Health Trust, Hammerfest, Norway; 3 Copenhagen Academy for Medical Education and Simulation (CAMES), Center for Human Resources and Education, Capital Region of Denmark, Copenhagen, Denmark; 4 Faculty of Health Sciences, Department of Quality and Health Technology, University of Stavanger, Stavanger, Norway; 5 Department of Public Health, Copenhagen University, Copenhagen, Denmark; 6 Capturo AS, Skjetten, Norway; 7 Faculty of Health Sciences, Department of Anesthesia, McMaster University, Hamilton, ON Canada

**Keywords:** NorMS-NTS, Nontechnical skills, Medical students, Assessment, Simulation-based training, Validation, Assessment tools

## Abstract

**Background:**

The NorMS-NTS tool is an assessment tool for assessing Norwegian medical students’ non-technical skills (NTS). The NorMS-NTS was designed to provide student feedback, training evaluations, and skill-level comparisons among students at different study sites. Rather than requiring extensive rater training, the tool should capably suit the needs of busy doctors as near-peer educators. The aim of this study was to examine the usability and preliminary assess validity of the NorMS-NTS tool when used by novice raters.

**Methods:**

This study focused on the usability of the assessment tool and its internal structure. Three raters used the NorMS-NTS tool to individually rate the team leader, a medical student, in 20 video-recorded multi-professional simulation-based team trainings. Based on these ratings, we examined the tools’ internal structure by calculating the intraclass correlation coefficient (ICC) (version 3.1) interrater reliability, internal consistency, and observability. After the rating process was completed, the raters answered a questionnaire about the tool’s usability.

**Results:**

The ICC agreement and the sum of the overall global scores for all raters were fair: ICC (3,1) = 0.53. The correlation coefficients for the pooled raters were in the range of 0.77–0.91. Cronbach’s alpha for elements, categories and global score were mostly above 0.90. The observability was high (95%-100%). All the raters found the tool easy to use, none of the elements were redundant, and the written instructions were helpful. The raters also found the tool easier to use once they had acclimated to it. All the raters stated that they could use the tool for both training and teaching.

**Conclusions:**

The observed ICC agreement was 0.08 below the suggested ICC level for formative assessment (above 0.60). However, we know that the suggestion is based on the average ICC, which is always higher than a single-measure ICC. There are currently no suggested levels for single-measure ICC, but other validated NTS tools have single-measure ICC in the same range. We consider NorMS-NTS as a usable tool for formative assessment of Norwegian medical students’ non-technical skills during multi-professional team training by raters who are new to the tool. It is necessary to further examine validity and the consequences of the tool to fully validate it for formative assessments.

## Background

Non-technical skills (NTSs) are defined as ‘the cognitive, social and personal resource skills that complement technical skills and contribute to safe and efficient task performance’ [1]. Examples of NTSs include skills in decision making, leadership, teamwork, situation awareness, etc. [2]. Studies show that NTSs can be improved through training [3–6]. Medical students need to learn NTSs during medical school, as the high-level use of NTSs is important for patient safety [1, 7]. Poor NTS performance has been identified as a contributing factor in 70% of the adverse events that occur in hospitals [8].

Training NTS requires an NTS assessment tool to ensure that medical students successfully obtain these skills during medical school. NTS tools can be used to evaluate students’ NTS performance, give them feedback and evaluate the NTS training. Several tools have been developed for the assessment of health professionals’ NTSs [9–14]. The most versatile and flexible is the Scottish Anesthetists Non-Technical Skills rating system (ANTS) [9]. This has been further developed into Danish and Norwegian adaptations aimed at assessing nurse anesthetists [15]. Other tools are the Non-Technical Skills for Surgeons (NOTSS) [16], Anesthetists Non-Technical skills for Anesthesia Practitioners (ANTS-AP) [17] and the Scrub Practitioners' List of Intraoperative Non-Technical Skills (SPLINTS) [18]. For medical students, the Medical Students’ Non-Technical Skills (Medi-StuNTS) [19] was created in the United Kingdom [20]. There is also a tool for anesthesiology students, the Anesthesiology Students’ Non-Technical skills (AS-NTS) [14].

There is evidence of the need to develop customized tools for each profession and even for specific countries and cultures [21, 22]. Different countries have differences in culture, tasks and responsibilities, which likely require contextualizing what NTS is about and how they would be used. Studies have found that NTS tools developed in the United Kingdom had to be adapted for use in a Danish setting [22]. To avoid a risk of overlooking specific desired NTS for Norwegian Medical students if adapting an existing tool. we decided to create a new tool to assess Norwegian medical students’ nontechnical skills (NorMS-NTS) [23]. The process of the development of NorMS-NTS has been thoroughly described previously [23].

NorMS-NTS was created as a tool for assessing NTS in relation to student feedback, training evaluations, and comparing student skills levels among different study sites. To facilitate a broader adoption of the tool and to optimize the validation of data, the ease of use was a critical feature for this tool. That the tool does not require extensive rater training was thus of importance.

The aim of this study was to examine the usability and preliminary assess validity of the NorMS-NTS tool when used by novice raters.

We recognize that validity interpretation is not simply a matter of either being valid or not [24]. The issue of validity is measured through scores, interpretation, and use, not simply by the tool. Different uses of the same tool may lead to diverging results. In other words, validity is context dependent. When validating NTS assessment tools, it is important to define and clearly specify the intended context. Evidence validated in one specific setting is often transferable to another setting, but that should be specifically determined according to each situation. Validation is a continuous process of collecting evidence over time and in different contexts.

As the aim of this first part of the validation process was to examine novices’ use of the NorMS-NTS. Our focus in this study is the usability of the tool and its internal structure, as measured by interrater reliability, internal consistency, and observability. A full validation for formative assessment with consequences and impact on students is beyond the scope of this article. We did not collect validity evidence for the use of the tool for summative assessment, as it requires extended rater training. Previous studies from aviation show that even those who know human factors need 2–3 days of training and calibration to reach sufficient single rater inter-rater reliability [25].

## Methods

The NorMS-NTS consists of four categories, 13 elements and an overall score (Table 1). The categories and elements are rated on a 5-point Likert scale, and the overall global scores are rated on a 7-point Likert scale.

**Table 1:** NorMS-NTS

Category^a^	Category score^b^	Element^a^	Element score^b^	Feedback
Communication		Team communication		
		Establish mutual understanding		
		Patient communication		
Situation awareness		Situational assessment		
		Understanding of team members’ roles		
		Attentiveness		
Teamwork		Professional modesty		
		Flexibility		
		Efficient use of team members		
Decision making		Uncertainty management		
		Decision analysis		
		Leadership		
		Prioritization		

General comments: _________________________________________________________________________________________________________________________________________________________________________________________________________________

^a^N/A – Not applicable. 1, much below average; 2, below average; 3, acceptable; 4, above average; 5, much above average

^b^Within team unless otherwise specified

Overall global rating (marked with a ring):

Very poor 1–2 – 3–4 – 5–6 – 7 Excellent

Validity evidence was collected by performing as an observational study using three raters to assess the human performance evidenced in 20 videos. Three doctors from RegSim were recruited as raters. RegSim is a unit at the Northern Norway Regional Health Authority (Helse Nord) that is responsible for simulation training in all hospitals in northern Norway. All three doctors had broad clinical experience and shared a stated interest in simulation (Table 2). The raters were blinded to the participants’ educational grade. The three raters were required to read the NorMS-NTS training manual developed by the author (KP). The research team member KP delivered a 20-min overview of the tool to all three raters via Microsoft Teams ®. The three raters were then given online access to the videos through an online data portal. Raters received the tool through e-mail. Each rater individually rated the team leader (medical student) through 20 video-recorded multiprofessional simulation-based team trainings using the NorMS-NTS tool. One rater completed the forms electronically and sent them to researcher KP via email. The remaining two raters printed the forms and filled them out manually, then they scanned them and returned them via e-mail.

**Table 2:** Raters’ backgrounds

Background	Rater 1	Rater 2	Rater 3
Age	*57*	*51*	*46*
Specialization	*Pediatrician*	*Anesthesiologist*	*Anesthesiologist*
Academic competency highest degree/position?	*PhD*	*Cand. med*	*Cand. med*
Clinical experience (number of years in clinical practice)	*30 years*	*25 years*	*19 years*
Do you have any prior experience with nontechnical skills (NTSs) or tools for NTS assessment?	*No*	*Yes*	*Yes, many years of experience with simulation training, but not with specific tools like this*

Each video was assigned a study identification number consisting of two digits, and the three raters were assigned the numbers 01, 02 or 03. The data from the raters’ marking sheets were entered into an Excel sheet. The data were then imported into the Statistical Analysis System (SAS^©^ ver. 9.4) for analysis. The data were checked for possible errors, such as incorrect scales or missing ratings. Then, the data were stored in a permanent and password-protected SAS database in preparation for the analyses.

### Setting

The medical students participating in this study were enrolled as students at UiT—The Arctic University of Norway in Hammerfest, Tromsø and Bodø. All students had multi-professional team training as part of their curriculum. The teams mostly consisted of medical students and nursing students, although some teams also had radiography students or bioengineering students on their team. The medical students were in their 5^th^ and 6^th^ years of study. Two different simulation-based training scenarios were used, and they were implemented following detailed descriptions in scenario scripts. Each simulation lasted between 12–20 min. Due to COVID-19 restrictions, some of the scenarios were implemented using a simulation manikin rather than a simulated patient. All scenarios had a trained nurse or doctor as the facilitator. The simulated patient was examined, answered student questions, and expressed pain and emotions. The students performed all measures and examinations, and the facilitator then informed them of the results consecutively. If the desired equipment was not available, the students were told to say what they would have done, which is a low-cost, easily accessible method of simulation training that can be performed anywhere.

### Ethics

Norwegian law exempts educational studies from ethical approval because they do not involve patients. However, the Regional Committee of North Norway for Medical and Health Research provided feedback on the protocol used in this study and approved this assumption (Ref: 2016/1539/REK nord). The participant consent form was approved by the Norwegian Center of Research Data (Ref: 57,474/2017). Informed consent from all participants was obtained after oral and written information was delivered on the purpose and objectives of the study.

The rating of the videos was performed on the Services for sensitive data (TSD) facilities owned by the University of Oslo, operated and developed by the TSD service group at the University of Oslo, IT department (USIT). All videos were saved at the TSD. TSD provides a platform for public research institutions in Norway. This service provides a secure project area where researchers can collect, store, and analyze sensitive data.

### Validity dimensions

Messick’s framework is recommended as a method of collecting evidence to validate assessment tools [24]. There are other frameworks available, but we chose Messick’s, as it has been the standard in the field since 1999 [26]. It is a conceptual, theoretical framework that utilizes five sources of evidence: content, internal structure, relationship with other variables, response process and consequences. We have summarized our validation procedures for different sources in Table 3, which displays the different dimensions we used to investigate validity evidence regarding the use of the NorMS-NTS.

**Table 3:** Messick framework: sources of evidence, definitions and procedure

Source of evidence	Definition	Procedure
**Content**	“the relationship between a test's content and the construct it is intended to measure [26].”	*Assessed as a part of development*
**Internal structure**	"The relationship among data items within the assessment and how these relate to the overarching construct [24]"	Interrater reliability
		Internal consistency
		Observability
**Relationships with other variables**	“The degree to which these relationships are consistent with the construct underlying the proposed test score interpretations [26]”	*Planned in further validations*
**Response process**	“The fit between the construct and the detailed nature of performance... actually engaged in [26]”	Raters respond in questionnaire
**Consequences**	“The impact, beneficial or harmful and intended or unintended, of assessment [27]”	Evaluation of the possibility of minimal rater training

### Content

Evidence for validation of the tool’s content was collected during the development of the NorMS-NTS [23]. The tool was created based on information gathered from focus group interviews. Participants in these focus groups provided their views regarding which NTS were necessary for newly graduated physicians. After analyzing the interviews, the participants were asked to provide feedback regarding the tool. Participants were asked if the tool accurately reflected their opinions and inputs. The feedback provided indicated that the assessment tool accurately reflected their opinions. Despite beginning the tool’s development from scratch, the tool was quite similar to previously described tools, demonstrating convergent validity and thus supporting content validity [9, 21, 28, 29].

### Internal structure

#### Interrater reliability

ICC (3,1) was calculated as all subjects were being rated by the same specific population of raters. The nonparametric statistic Kendall’s W was also used to assess the level of agreement between raters.

#### Internal consistency analysis

The correlation between the elements, categories and overall global scores was measured. The Spearman nonparametric correlation between each category and the corresponding elements was calculated, as well as that between the global scores and the categories. In addition, Cronbach’s alpha (CA) was applied.

#### Observability

The observability of each element, category and global score was calculated by the percentage of observations recorded by the raters. An observability > 50% is deemed acceptable [30].

### Response process

All raters received a questionnaire after they had completed rating all of the videos (Table 4). Raters were asked to give feedback on the tool, including whether they found it to be unclear, difficult to use, or any other inputs. The answers are summarized completely in Table 4.

**Table 4:** Raters questionnaire

Background:
Age:
Specialization:
Academic competency highest degree/position?
Clinical experience (number of years in clinical practice):
Do you have any prior experience with nontechnical skills (NTS) or tools for NTS assessment?
Usability of the tool:
How was the tool to use?
How easy was it to assess the students' skills in elements and categories?
Were there elements of nontechnical skills that the tool did not capture?
Were there elements that you felt were redundant, i.e., should not have been included in the tool?
Were there elements that were difficult to assess?
Were the written instructions helpful?
Did you find that it became easier or more difficult to use the tool after gaining more experience with its use?
How long did you spend on average rating the videos?
Is this a tool you could use for training or teaching?
Other feedback?

### Consequences

We examined the possibility of using NorMS-NTS after minimal rater training. For a high-stake summative assessment, an ICC of above 0.70 is suggested [31]. For a formative assessment, a minimum ICC is not clearly specified. An ICC above 0.60, however, is proposed [31]. The proposed ICC levels are based on the average ICC. The average ICC levels are always higher than the single-measure ICCs [32]. We could not find any proposed levels for single ICC measures for formative assessment.

## Results

The average overall global scores for the three raters across the 20 videos was 4.7 (SD = 1.1), 4.3 (SD = 1.4) and 4.0 (SD = 2.0).

### Internal structure

#### Interrater reliability

An ICC below 0.40 is considered as a poor correlation, between 0.40 and 0.59 is considered a fair correlation, between 0.60 and 0.74 is considered an good correlation and between 0.75 and 1.00 as excellent correlation [33]. The ICC agreement for the sum score of the overall global score for all raters was fair: ICC (3,1) = 0.53 [33]. This was supported by Kendall’s W = 0.73 (Table 5). Two of the raters had a higher level of experience, and once an agreement analysis for those two only was applied, the level of agreement was higher. ICC (3,1) = 0.53 was still fair [33]; however, Kendall’s W = 0.80 was good. The individually calculated ICC (3,1) and Kendall’s W are both lower (0.25–0.55 and 0.51–0.75, respectively).

**Table 5:** Inter-rater agreement statistics. ICC and Kendall’s W

	**All raters**		**Rater 2 and 3**	
**Score**	**ICC(3,1)**	**Kendall’s W**	**ICC(3,1)**	**Kendall’s W**
*Communication*	0.49	0.69	0.37	0.71
Team communication	0.43	0.63	0.48	0.77
Establish mutual communication	0.55	0.75	0.45	0.80
Patient communication	0.54	0.68	0.45	0.74
*Situational awareness*	0.50	0.69	0.43	0.73
Situational assessment	0.27	0.51	0.07	0.56
Understanding of team members’ roles	0.39	0.63	0.13	0.58
Attentiveness	0.44	0.68	0.37	0.76
*Teamwork*	0.40	0.62	0.20	0.63
Professional modesty	0.25	0.51	0.02	0.55
Flexibility	0.41	0.67	0.40	0.76
Efficient use of team members	0.40	0.62	0.25	0.64
*Decision making*	0.44	0.68	0.49	0.79
Uncertainty management	0.36	0.57	0.46	0.75
Decision analysis	0.43	0.61	0.58	0.81
Leadership	0.49	0.72	0.48	0.82
Prioritization	0.33	0.56	0.37	0.71
*Overall Global Score*	0.53	0.73	0.55	0.80
*Sum of communication elements*	0.58	0.76	0.51	0.81
*Sum of situational awareness elements*	0.41	0.67	0.21	0.67
*Sum of teamwork elements*	0.42	0.68	0.28	0.71
*Sum of decision-making elements*	0.46	0.66	0.55	0.82
*Sum of all elements*	0.50	0.72	0.45	0.82
*Sum of categories*	0.52	0.73	0.45	0.80

#### Internal consistency analysis

For both the Spearman correlation coefficient and Cronbach’s alpha, a correlation coefficient of near 1.0 represents high internal consistency. Most of the Spearman correlations were above 0.80 (Table 6). The correlation coefficients for the pooled raters were in the range of 0.77–0.91. Almost all correlation coefficients were significant at the *p* = 0.0001 level. Cronbach’s alpha for the elements, categories and global scores were all mostly above 0.90, which is in the excellent range and thus confirms a high level of scoring consistency among the raters.

**Table 6:** Consistency in scoring by Spearman correlation coefficient for category vs. elements or global score vs. categories

	**Spearman correlation coefficient for category vs. elements or global score vs. categories**				**Cronbach’s alpha (standardized variables)**			
**Score**	**Rater 1**	**Rater 2**	**Rater 3**	**Raters pooled**	**Rater 1**	**Rater 2**	**Rater 3**	**Raters pooled**
*Communication*	-	-	-	-	0.94	0.74	0.90	0.92
Team communication	0.88	0.85	0.89	0.91	0.96	0.75	0.92	0.93
Establish mutual communication	0.89	0.68	0.85	0.86	0.96	0.84	0.92	0.94
Patient communication	0.90	0.43	0.77	0.82	0.95	0.87	0.96	0.95
*Situational awareness*	-	-	-	-	0.95	0.52	0.98	0.94
Situational assessment	0.96	0.47	0.91	0.88	0.95	0.84	0.98	0.96
Understanding of team members’ roles	0.88	0.82	0.91	0.87	0.97	0.66	0.98	0.96
Attentiveness	0.93	0.58	0.96	0.89	0.96	0.74	0.97	0.95
*Teamwork*	-	-	-	-	0.92	0.77	0.97	0.94
Professional modesty	0.85	0.71	0.97	0.88	0.94	0.80	0.98	0.95
Flexibility	0.92	0.52	0.97	0.88	0.93	0.88	0.98	0.95
Efficient use of team members	0.87	0.73	0.95	0.89	0.96	0.79	0.99	0.96
*Decision making*	-	-	-	-	0.94	0.88	0.97	0.95
Uncertainty management	0.88	0.85	0.89	0.87	0.95	0.88	0.97	0.95
Decision analysis	0.88	0.76	0.82	0.80	0.96	0.91	0.98	0.96
Leadership	0.92	0.57	0.92	0.86	0.95	0.94	0.97	0.96
Prioritization	0.87	0.90	0.95	0.90	0.95	0.89	0.97	0.95
*Overall global score*	-	-	-	-	0.94	0.82	0.98	0.95
*Communication*	0.88	0.82	0.90	0.81	0.96	0.84	0.98	0.95
*Situational awareness*	0.93	0.74	0.89	0.77	0.95	0.85	0.98	0.95
*Teamwork*	0.86	0.55	0.94	0.79	0.97	0.90	0.98	0.95
*Decision making*	0.94	0.69	0.93	0.86	0.95	0.86	0.98	0.95

#### Observability

Observability was calculated as the percentage of elements and categories that were not scored with n/a. Two of the marking forms had completed scoring of all elements scored but not all categories. This was considered an error, as all elements were observed. Those two forms were not included in the statistics. The observability was deemed acceptable (95%-100%) (Table 7).

**Table 7:** Observability

Elements	Observability
Team communication	100%
Establish mutual understanding	100%
Patient communication	100%
Situational assessment	100%
Understanding of team members’ roles	100%
Attentiveness	100%
Professional modesty	100%
Flexibility	100%
Efficient use of team members	100%
Decision analysis	97%
Uncertainty management	95%
Leadership	98%
Prioritization	100%
**Categories**	
Communication	100%
Situational awareness	100%
Teamwork	100%
Decision making	99%

### Response process

The raters’ responses are summarized in Table 8. All the raters found the tool easy to use, none of the elements were identified as redundant, and the written instructions were helpful. Raters also found the tool easier to use once they gained practice in using it. Raters with NTS experience had a shorter time of use per video than the novel rater. All the raters stated that they could use the tool for training or teaching.

**Table 8:**
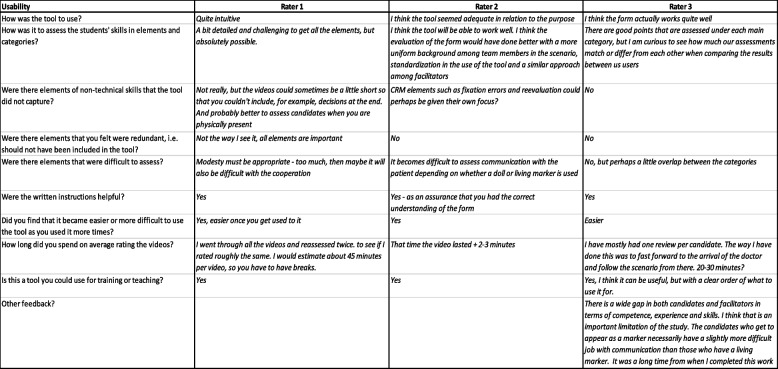
Rater feedback

Some of the videos were reported to be slightly too brief to properly assess all elements for scoring. One of the raters suggested that the ratings should have been more standardized, that team members should be more uniform and that facilitators should take a similar approach. It was also mentioned that communication depended on whether the patient was a manikin or a simulated patient. One rater suggested that crew resource management (CRM) elements, such as fixation errors and reevaluations, could be given a greater focus in the tool.

### Consequences

The calculations show that the use of NorMS-NTS by raters new to the tool reaches an ICC of 0.53. That value is 0.08 below the suggested ICC level for formative assessment of above 0.60 [34].

## Discussion

The NorMS-NTS tool was developed for the assessment of Norwegian medical students’ nontechnical skills. Our aim has been to create an easy-to-use tool that suits busy doctors as near-peer educators in both clinical teaching settings and during simulation-based training. Ideally, this tool should be easy to find online, and raters should be able to use the tool after only a short introduction. The interpretation of the validation results described in this study was based on these principles.

The raters found the tool usable. They found all the categories and elements relevant. The raters considered the written instructions helpful. We will improve them further, especially for the categories and elements with the lowest ICC. All raters could use the tool for training or teaching. The least experienced rater used 45 min to rate videos, which is not feasible in clinical practice, bu the experienced raters used only a few minutes more than the duration of the scenario. Therefore, raters will probably be more efficient as they become accustomed to the tool. The raters also described that in their feedback. The internal structure of the tool was excellent. The observability was also found to be excellent. These findings support the tools’ structure and content. The usability of the tool was found to be satisfactory.

The usability for the raters after only a short introduction is an important part of the ‘Consequences’. On the other hand, the consequences for the students are also important to investigate further. Such studies should explore the students’ views. Are they assessed fairly? Do they get ideas for improvement? Does the assessment motivate or encourage them? It is also important to explore the system consequences. Is it possible to integrate such a tool in education? Do teachers and learners use the tool to clarify learning potential, or a test to pass or fail. Do we have the tools to help those who struggle? This is all out of scope for this paper but should be studied further.

The individual interrater reliability after a short introduction and training was found to be fair. We found a single measure ICC of 0.53 for the global overall score. That ICC is 0.08 below the suggested ICC level for formative assessment (above 0.60) [34]. However, we know that the suggestion is based on the average ICC, which is always higher than a single-measure ICC [35]. Comparing to other NTS tools, ICC is challenging, as the ICC calculations are not specified [36]. In studies where single-measure ICC is calculated with raters novice to the tool the findings are quite similar to ours. The NOTSS single measure ICCs on the category scores varied from 0.29 to 0.66 [37]. The Medi-StuNTS reached a single-measure ICC of 0.37 [36]. Other studies where ICC is not specified as single-measures or average the ICC are still in the same range as NorMS-NTS [38]. A study comparing ANTS and Ottawa GRS found ICCs of 0.39 and 0.42 for overall scores [39]. As there are no suggested levels for single-measure ICCs for formative assessment for novice raters [36], we consider the calculated levels to be sufficient for conducting a formative assessment of medical student NTS, as they are in the same range as for other validated NTS tools. The average ICC (3.1) would be more appropriate to use for validation for summative assessment and should be applied in later validations of the tool.

There are several ways to increase interrater reliability, i.e., rater training, modification of the assessment tool, stricter scenario design, etc. Previous studies have shown that the level of interrater agreement increases when raters gain more experience with an assessment tool [40]. As the NorMS-NTS is usable with minimal training, it is also possible for busy doctors to gain experience with the tool, hence increasing its interrater reliability. We will also continue to refine the NorMS-NTS training introduction and training manual in the areas that were identified as poor.

### Limitations

As collecting validity evidence of NTS assessment tools is a continuous process of collecting evidence of validity, this article only describes part of the validation necessary to meet all accepted sources of evidence in the Messick framework. We have tried to clearly specify the context and intended use we have assessed usability and preliminary validation of NorMS-NTS for in this article. We did not seek validity evidence of the use of the tool for summative assessment with minimal rater training now. Further collection of validity evidence as described in the Messick framework is planned, including for summative assessment using average ICC. To fully validate the tool for formative assessment, it is necessary to further study the consequences of the tool. That is, we explore the impact on the students and see if the formative assessments obtained by the tool are correct and beneficial.

The raters had some input about the validation process itself. We deliberately chose to not have standardized scenarios, teams, and facilitators. We wanted a tool that works in everyday life, with different facilitators, team members and situations. All raters rated the same scenarios in the study, so they had the same variety. We would probably have achieved a higher level of interrater reliability with a greater degree of standardization of the scenarios and ratings, but the findings may not have been transferable to practical use. Some suggest that all validation of assessment tools should include true measurers of validity and reliability, and we have worked to achieve this in our study [41].

As this preliminary validation process was created to validate the tool for formative assessment for busy doctors as near-peer educators in clinical practice, we chose single-measure ICCs. Because of that, we only had three raters. When validating the tool for summative assessment, more raters will be included.

The tool was developed in Norway. When using it in different contexts, be it different places within Norway or in different countries, pilot studies should be conducted, collecting context-specific validity evidence again. Using such a tool and interpreting its results is a complex socio-technical endeavor with possible consequences for healthcare professionals and the people who they treat. Therefore, it seems appropriate to double check.

## Conclusions

We collected preliminary evidence of validity for the NorMS-NTS tool. Raters found the tool usable. When the NorMS-NTS was used by raters new to the tool we found that the interrater reliability, internal consistency, and observability were sufficient for formative assessment. It is necessary to further examine the consequences of the tool to fully validate the tool for formative assessment.

### Further

The process of validation for the NorMS-NTS began with this study. A summative assessment study calculating the average ICC is planned for the future. Further validation should focus on the final two sources of evidence in the Messick framework: relationship with other variables and consequences. We note that it is also important to validate the tool for different settings.

## Data Availability

The datasets used and/or analyzed during the current study are available from the corresponding author upon reasonable request.

## Abbreviations

NorMS-NTS: Norwegian Medical Student’s Non-technical Skills
NTS: Non-technical skills
TSD: Services for sensitive data
